# *Akkermansia muciniphila* administration exacerbated the development of colitis-associated colorectal cancer in mice

**DOI:** 10.7150/jca.63578

**Published:** 2022-01-01

**Authors:** Fei Wang, Kuntai Cai, Qiuxiang Xiao, Lihua He, Lu Xie, Zhiping Liu

**Affiliations:** 1 Gannan Medical University, Ganzhou, Jiangxi, 341000, China.; 2 The Fifth People's Hospital of Jinan, Jinan, Shandong, 250000, China.; 3 School of Basic Medicine, Gannan Medical University, Ganzhou, Jiangxi 341000, China.; 4 Center for Immunology, Key Laboratory of Prevention and Treatment of Cardiovascular and Cerebrovascular Diseases, Ministry of Education, Gannan Medical University, Ganzhou, Jiangxi 341000, China.

**Keywords:** *Akkermansia mucinipila*, Colorectal cancer, Cell proliferation, Inflammatory cytokines

## Abstract

Colorectal cancer (CRC) is one of the most common digestive tract malignancies and inflammation and gut microbiota are well-known key factors to influence CRC development. *Akkermansia mucinipila* is an important gram-negative anaerobic bacterium that can degrade mucin in gut. Previous studies suggested that *A. muciniphila* may affect inflammation and cell proliferation, but the relationship between *A. muciniphila* and CRC is not clarified. Here C57BL/6 mice were administrated with *A. muciniphila* or PBS and then treated with azoxymethane (AOM)/dextran sodium sulphate (DSS) to induce CRC. The mice receiving *A. muciniphila* administration had more serious weight loss, shorter colon length and more intestinal tumors than those receiving PBS administration after AOM/DSS treatment. More colon damage and less goblet cells were also observed in *A. muciniphila* treated mice. Furthermore, *A. muciniphila* administration induced more Ki67^+^ proliferating cells, higher PCNA expression and elevated gene expression of proliferation-associated molecules including *Snrpd1*, *Dbf4* or *S100A9*. At early stage of CRC development, in comparison with controls, the mice receiving *A. muciniphila* administration also had more body weight loss and shorter colon length, as well as higher gene expression of inflammatory cytokines. Furthermore, the *in vitro* experimental results showed that the co-culture of colon epithelial cells with *A. muciniphila* enhanced the cell proliferation and gene expression of proliferation-associated molecules. Therefore, *A. mucinipila* may promote the formation of CRC in mice through increasing the early level of inflammation and the proliferation of intestinal epithelial cells.

## Introduction

Colorectal cancer (CRC) is one of the most common digestive tract malignancies and a major cause of cancer-related morbidity and mortality, which has done great harm to people's health around the world 1. Despite its severity, the pathogenesis of CRC is still unclear, and the current treatment has not been quite satisfying. Therefore, the studies on the molecular mechanism of CRC development that can provide new ideas and therapeutic targets for its diagnosis and treatment are in urgent need. The cause of CRC is complicated and involves genetic factors, diet, and inflammation. Among them, inflammation is a well-known risk factor. Clinically, patients with inflammatory bowel diseases (IBD) had an increased risk of developing CRC, which was also supported by the studies on CRC pathogenesis utilizing IBD mouse model 2, 3.

Absent in melanoma 2 (AIM-2) is a pattern recognition receptor that can recognize dsDNA. In our previous study and other studies, it was found that AIM2 could suppress colitis-associated colorectal cancer (CAC) induced by azoxymethane (AOM) and dextran sodium sulfate (DSS) treatment. The underlying mechanism can be divided into two parts: on the one hand, AIM2 suppressed overt proliferation by controlling the proliferation of intestinal epithelial cells as well as the expansion of intestinal stem cells 4-6. On the other hand, AIM2 suppressed colorectal tumorigenesis by modulating the gut microbiota 6. The genomic sequencing of mouse microbiota showed that *Aim2^-/-^* mice harbored increased levels of *Akkermansia muciniphila*, *Anaeroplasma*, and decreased levels of *Anaerostipes*, *Bifidobacterium*, *Flexispira*, *Prevotella* and* Paraprevotella* species relative to WT mice. More importantly, the susceptibility to CRC could be reduced in *Aim2^-/-^* mice by co-housing them with WT mice, indicating that WT mouse might harbor some protective microbiota in their intestine, and there's potential gut microbiota alteration in *Aim2^-/-^* mice which led to hyper-susceptibility to CRC 6. However, it's still unclear how the alteration of gut microbiota affected the proliferation of intestinal stem cells as well as the progression of CRC.

*A. muciniphila*, a gram-negative anaerobic bacterium from phylum* Verrucomicrobia*, can be isolated from the human intestine and other animals. It was previously shown that *A. muciniphila* can produce acetate and propionate during its growth on mucin 7, 8. *A. muciniphila* engraftment in the mucosal layer of intestinal mucosa triggers the host's metabolic and immune responses. The number of *A. muciniphila* is often considered as an indicator in monitoring host's metabolic status due to the bacterium's ability to increase the thickness of intestinal mucosa and to enhance the intestinal barrier function. Other than that, *A. muciniphila* can produce short-chain fatty acids (SCFAs) that are in favor of the host and other gut microbiota, thus improving intestinal homeostasis 9.

The relationship between *A. muciniphila* and CRC remains controversial. A study showed that the abundance of *A. muciniphila* in CRC patients was 4 times higher than that of healthy subjects 10. In addition, *A. muciniphila* was also found to be significantly increased in mucosal biopsy samples of CRC patients 11. Another study showed that the number difference of *A. muciniphila* between CRC patients and non-CRC patients was indistinctive 12. In the meantime, it was showed decreased number of *A. muciniphila* in CRC patients with severe symptoms, indicating that *A. muciniphila* did not contribute to CRC development 12, 13. As for animal studies, it was found that enrichment of *A. muciniphila* in mice developed CAC 14. Interestingly, a study found that mice with intestinal epithelial cells (IEC)-specific deficiency in Pten, a potent tumor suppressor, did not spontaneously develop CRC, which was correlated with diminished *A. muciniphila*, indicating the role of *A. muciniphila* in promoting CRC 15. Recently, a study showed that the treatment of vitamin D can alleviate CRC development and gut microbiota sequencing results showed increased number of *A. muciniphila* in mice with less symptoms of CRC 16. In conclusion, it is still baffling how the number of *A. muciniphila* affect CRC development.

Some other studies used oral administration of *A. muciniphila* methods to determine the role of *A. muciniphila* more directly in intestinal inflammation and tumorigenesis. During *Salmonella typhimurium* infection, *A. muciniphila* were shown to exacerbate *S. typhimurium*-induced intestinal inflammation with reduced goblet cell numbers 17. After introducing *A. muciniphila* to intestine specific conditional Apc mutant mice that spontaneously develop tumors in the colon, significantly increased tumor burden was observed in those mice, suggesting that *A. muciniphila* might promote tumorigenesis in the colon 18. These results suggest that *A. muciniphila* may promote inflammation and tumorigenesis in the intestine. However, it is still unclear whether or how *A. muciniphila* influences CRC development.

In our study, mice were orally gavaged by *A. muciniphila* before AOM/DSS treatment to develop CAC, then we evaluated the inflammation status and neoplastic development of those mice. *In vitro* study, *A. muciniphila* and human CRC cell line were co-cultured, then we monitored the influence of bacterium on cell proliferation. This study will contribute to our understanding on host interaction with specific intestinal bacteria and provide new ideas and theoretical basis for the prevention and treatment of CRC.

## Materials & Methods

### Mice

C57BL/6 mice were from Nanjing Animal Model Center (Nanjing, Jiangsu, China). They were maintained in a specific pathogen-free facility in the Experimental Animal Center of Gannan Medical University (Ganzhou, Jiangxi, China). This study was approved by the Ethics Committee of Gannan Medical University.

### Bacterial infection

*A. muciniphila* (ATCC#BAA-835) was grown anaerobically in brain heart infusion broth overnight and then sub-cultured. The bacterial solution was collected, and then diluted to a concentration of 1×10^8^ cells of *A. muciniphila* per 100 µl medium. The mice were orally administrated with PBS or *A. muciniphila* at the 0, 3, 5, and 7 days after experiments were initiated, and the feces were collected on day 0 and day 14 to validate the colonization of *A. muciniphila*.

### CAC animal model

The CAC animal model was established as previously described 6. Mice were intraperitoneally injected with AOM (10 mg/kg) and then treated with 3% DSS in drinking water for 5 days at 5 days after AOM injection. The mice were treated with DSS for another two rounds and sacrificed on the 80^th^ day after AOM injection to observe the tumor burden.

### RT-qPCR

Total RNA was extracted using Trizol (Invitrogen) following manufacture's instruction, and RNA concentration was measured using Nanodrop. mRNA was reversely transcribed into cDNA. qPCR was performed on an QuantStudio 7 Flex real-time PCR instrument with SYBR Green kit (ThermoFisher, Fremont, CA, USA) using corresponding primers (the sequences of the target gene were shown in **Supplementary Table 1**). The gene expression was quantitated by PCR with gene-specific primers. The relative gene expression was expressed as 2^-ΔΔCT^.

### Western-blot

Proteins were extracted from colon tissues using RIPA lysis buffer with protease inhibitors. The samples were resolved in SDS-PAGE and transferred onto NC membranes, Membranes were blocked in 5% milk for 1 hour and then incubated with anti- PCNA (CST, #13110, 1:1000 dilution) and anti-β-actin (Proteintech, #66009-1-Ig, 1:1000 dilution) antibodies overnight at 4 °C. After wash, membranes were incubated with Anti-rabbit (BOSTER, #BA1054) or anti-mouse (BOSTER, #BA1050) secondary antibody at room temperature for 1h. Finally, after adding ECL (Thermo, #A38555) substrates, the protein bands were analyzed by Chemdoc (Bio-rad).

### Hematoxylin & Eosin (H&E) staining and Immunohistochemistry

Colon tissues were embedded in paraffin, sectioned (4 μm), and stained using regular H&E methods. The inflammatory cells per crypt in slides were counted under the light microscopes. Histological lesion were scored by a histologist blindly based on the extent and severity of inflammation, ulceration, and hyperplasia of the mucosa as previously described 19. Specifically, scores for inflammation were as follows: 0 = normal (within normal limits); 1 = mild (small, focal, or widely separated, limited to lamina propria); 2 =moderate (multifocal or locally extensive, extending to submucosa); 3 = severe (transmural inflammation with ulcers covering >20 crypts). Scores for ulceration were as follows: 0 = normal (no ulcers); 1 = mild (1-2 ulcers involving up to a total of 20 crypts); 2 = moderate (1-4 ulcers involving a total of 20-40 crypts); 3 = severe (>4 ulcers or over 40 crypts). Scores for Mucosal hyperplasia were as follows: 0 = normal (within normal limits); 1 = mild (crypts 2-3 times normal thickness, normal epithelium); 2 = moderate (crypts 2-3 times normal thickness, hyperchromatic epithelium, reduced goblet cells, scattered arborization); 3 = severe (crypts >4 times normal thickness, marked hyperchromasia, few to no goblet cells, high mitotic index, frequent arborization). Scoring for extent of lesions: 0 = normal (0% involvement); 1 = mild (up to 30% involvement); 2 = moderate (30%-70% involvement); 3 = severe (over 70% involvement).

Paraffin-embedded tissue sections were re-hydrated, and their antigen retrieval was performed using citric acid sodium buffer. Sections were then incubated overnight at 4 °C with a primary antibody targeting the Ki67 (Sigma, #SAB4501880, 1:800 dilution). Sections were then washed and subsequently incubated with goat anti-rabbit IgG-peroxidase (Sigma, #A9169, 1:1000 dilution) for 30 min at room temperature. After wash, the sections were stained with DAB. Finally, the sections were dehydrated, made transparent in xylene, and mounted with neutral gum. The Ki67^+^ cells per crypt in slides were counted under the light microscopes.

### AB-PAS staining

This was performed using AB-PAS staining solution kit (Servicebio, Wuhan, China). Specifically, tissues sections were rehydrated, then stained with PAS dye solution C and B for 15 min, respectively. Then sections were then rinsed with running and distilled water. After that, PAS dye solution A was used to stain the sections. Finally, the sections were dehydrated, made transparent in xylene, and mounted with neutral gum. Then the stained goblet cells per crypt were counted under the light microscopes.

### MTT assay

MTT kit (Solarbio, #M1020) were utilized. Human colon epithelial cell line SW620 (ATCC, # CCL-227) cells were collected and plated in 96-well plates. At day 1, 2, and 3, cells were incubated with 10^6^ *A. muciniphila* for 4 hours, then treated with gentamicin (Solarbio, Beijing) to kill extracellular bacteria, and further washed. After that, cells were then incubated with MTT solution for 4h, washed, and incubated with Formazan solution. Finally, OD_490_ values were measured.

### Statistical analysis

Data were analyzed using Graphpad Prism 7.0 software. Data were expressed as Mean ± SEM. The comparison among multiple groups and between two groups were performed by analysis of variance (ANOVA) and unpaired t-test, respectively. P<0.05 was considered statistically different.

## Results

### *A. muciniphila* administration increased host susceptibility to AOM/DSS induced CRC

**Figure 1:**
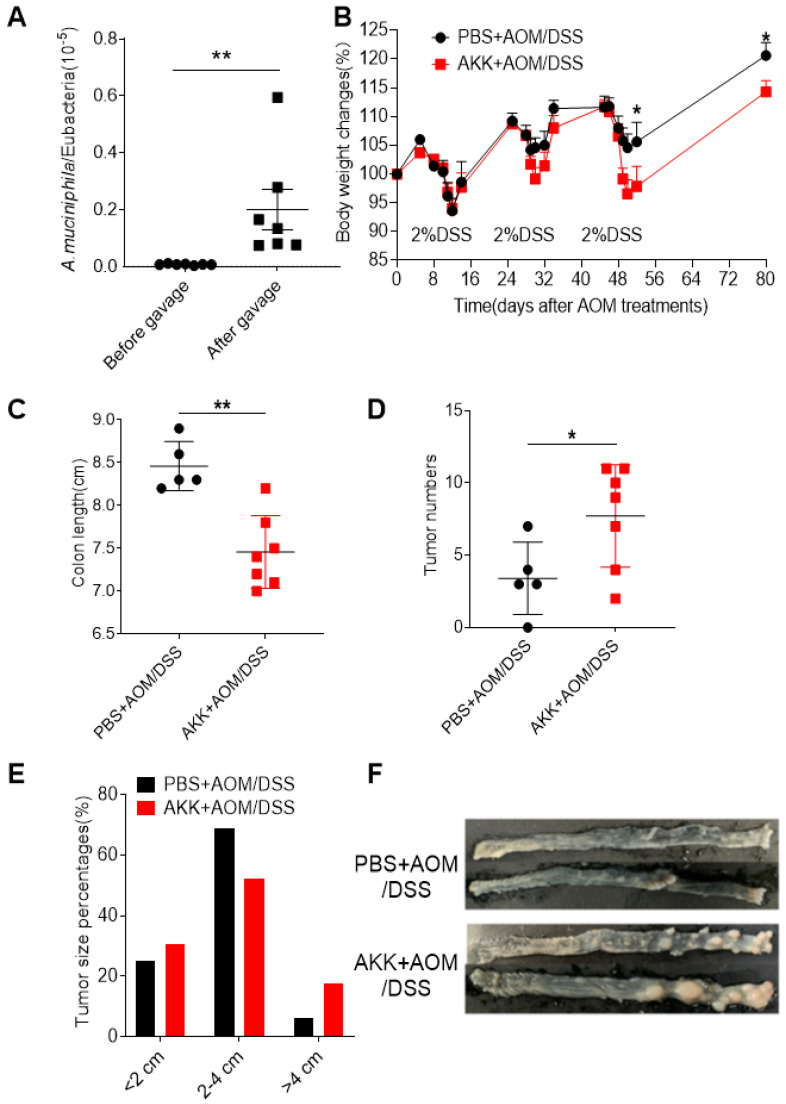
***Akkermansia muciniphila* administration enhanced the susceptibility of mice to colitis-related colorectal cancer (CAC).** C57BL/6 mice were orally administrated with *A. muciniphila* or PBS and treated with AOM/DSS to induce CRC. Bacterial colonization was validated in the feces collected before and after gavage by quantitative PCR. Mouse body weights were monitored during the AOM/DSS treatment. After 80 days, the mice were sacrificed, and the colons were collected to measure the tumor burden. **A.** The number of *A. muciniphila* in the feces of mice before and after gavage; **B.** body weight change; **C.** Colon length; **D.** The number of colon tumors; **E.** Tumor diameter; **F.** Representative photos of colon tumors. The data of A-D were represented by the Mean ± SEM, PBS+AOM/DSS, n=5; AKK+AOM/DSS, n=7. *P<0.05, **P<0.01, A, C, D. Unpaired student T test, B.Two-way ANOVA analysis. The experiments were repeated twice independently.

Gut microbiota plays a key factor in CRC development, but the role of *A. muciniphila* remains elusive. To directly assess the role of *A. muciniphila* in CRC, we orally inoculated mice with *A. muciniphila* or PBS before induction of CRC. Firstly, the feces were collected before and after the *A. muciniphila* inoculation, and DNA were extracted. The numbers of *A. muciniphila* were determined by qPCR using bacterium specific primers to validate the bacterial colonization. The results showed that the number of *A. muciniphila* in the feces significantly increased after *A. muciniphila* inoculation (**Figure 1A**), showing that *A. muciniphila* had been successfully colonized in the experimental mice. After that, AOM/DSS treatment were used to induce CRC and the body weight change was monitored. The AKK+AOM/DSS group mice that received *A. muciniphila* inoculation and AOM/DSS treatment lost more body weights on second and third rounds of DSS treatment than PBS+AOM/DSS group mice that received PBS inoculation and AOM/DSS treatment (**Figure 1B**). At 80 days post-AOM treatment, it was found that the colons of the mice in AKK+AOM/DSS group were significantly shorter than those in the PBS+AOM/DSS group (**Figure 1C**), and the mice in AKK+AOM/DSS group developed significantly increased numbers and size of tumors compared to mice in PBS+AOM/DSS group (**Figure 1D-F**). These results suggesting that *A. muciniphila* administration increased host susceptibility to AOM/DSS induced CRC.

### *A. muciniphila* promoted inflammatory cell infiltration, reduced the number of goblet cells, and promoted the proliferation of intestinal epithelial cells

**Figure 2:**
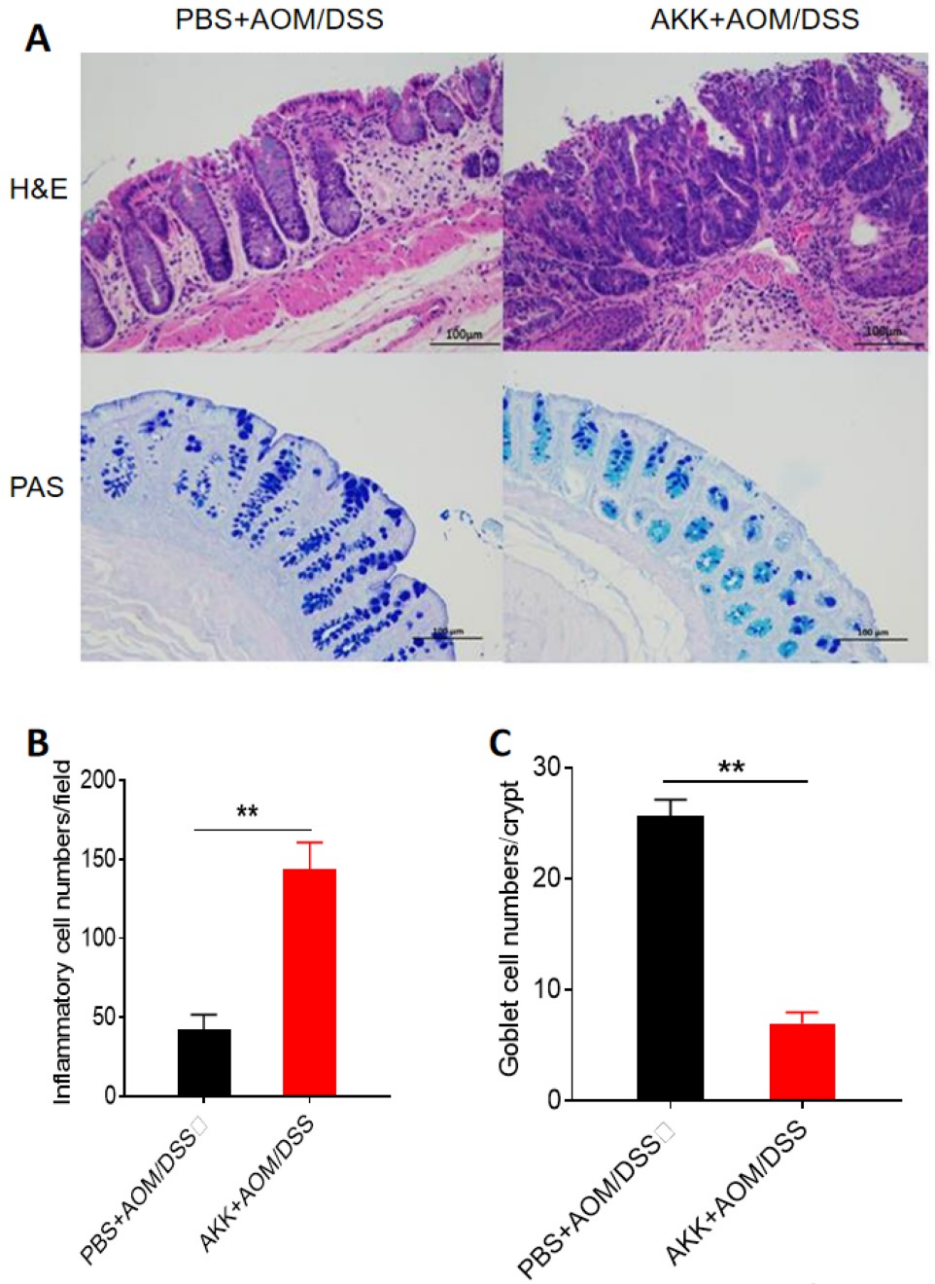
** Mouse colon tissue damage and goblet cell staining after *A. muciniphila* administration.** C57BL/6 mice were orally administrated with *A. muciniphila* or PBS and treated with AOM/DSS to induce CRC. The mice were sacrificed on the 80^th^ day after AOM treatment, and the colons were collected for histochemical staining. **A.** H&E staining and PAS staining, scale bar: 100µm; **B.** The average numbers of inflammatory cells in each crypt. **C.** The average number of goblet cells in each crypt. **P<0.01, B, C. Unpaired student T test. The experiments were repeated twice independently.

To further evaluate the tissue damage, we performed H&E staining in the colons at 80 days post AOM treatment. The AKK+AOM/DSS group mice presented more tissue damage and infiltration of inflammatory cells in the colons than PBS+AOM/DSS group mice (**Figure 2A, B**). *A. muciniphila* can degrade mucin mainly secreted by goblet cells in the intestine, and we performed staining analysis of goblet cells on colon tissues. The results showed that the goblet cells number in the AKK+AOM/DSS group was significantly reduced in comparison with the controls (**Figure 2A, C**).

**Figure 3:**
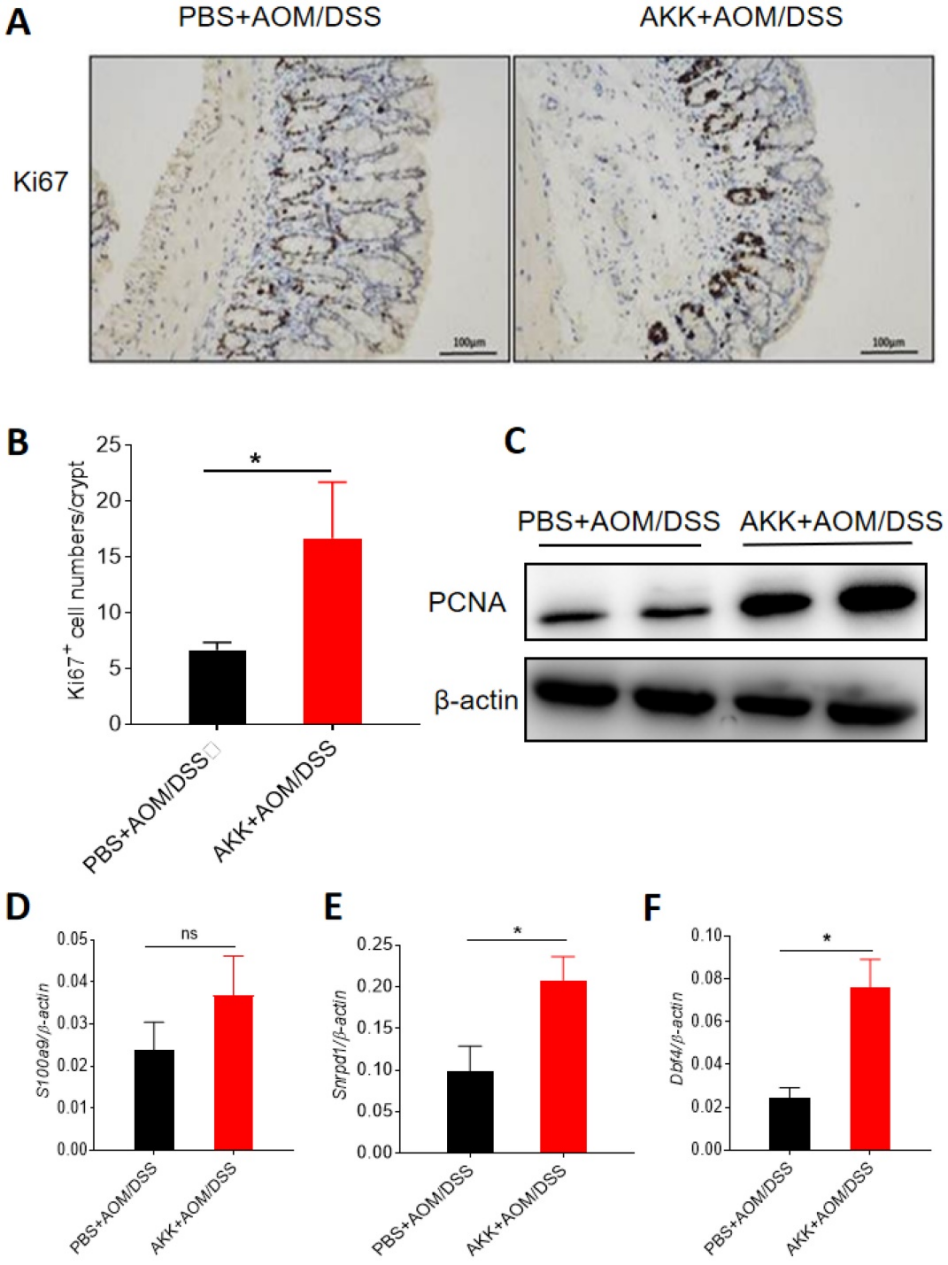
** Proliferation of mouse colonic cells was increased after *A. muciniphila* administration.** C57BL/6 mice were orally administrated with *A. muciniphila* or PBS and treated with AOM/DSS to induce CRC. The mice were sacrificed at day 80, and colon tissues were collected. Ki67 staining was applied using IHC method, and RT-qPCR and Western-blot were used to measure the expression of proliferation-related molecules. **A.** Ki67 staining results, scale bar: 100 um; **B.** The average numbers of Ki67+ cells in each crypt. **C.** Immunoblot analysis of PCNA in colon tissues; **D.** Colonic *S100a9* gene expression; E, *Snrpd1* gene expression. F, *Dbf4* gene expression. (C-E) Data were represented by Mean ± SEM. PBS+AOM/DSS, n=5; AKK+AOM/DSS, n=7. *P<0.05, ns: no significantly different, **B, D, E-F.** Upaired student T test. The experiments were repeated twice independently.

To further study the role of *A. muciniphila* colonization in the pathogenesis of CRC, we investigated the proliferation of the intestinal epithelial cells through immunohistochemical staining for Ki67. The numbers of Ki67-positive cells in the colons of AKK+AOM/DSS group were significantly higher than those in PBS+AOM/DSS group, indicating that *A. muciniphila* can promote the proliferation of intestinal epithelial cells (**Figure 3A-B**). Consistently, we measured the expression of PCNA, a marker for cell proliferation, in colon samples by Western-blot, and the results showed that AKK+AOM/DSS group had a higher level of PCNA protein expression than controls (**Figure 3C**). Calprotectin (S100 calcium binding protein A9, S100a9), small nuclear ribonucleoprotein D1 polypeptide (Snrpd1) and Dbf4 zinc finger protein (DBF4 zinc finger, Dbf4) are molecules associated with the proliferation of intestinal epithelial cells^6^. We used RT-qPCR to analyze the expression levels of these genes, and the results showed that the colonic expression level of *Snrpd1* and *Dbf4* were significantly elevated in AKK+AOM/DSS group relative to PBS+AOM/DSS group. The gene expression of S100a9 tended to increase in AKK+AOM/DSS group as well (**Figure 3D-F**). These results suggest that *A. muciniphila* can promote the proliferation of intestinal epithelial cells.

### *A. muciniphila* administration aggravated inflammation in mice early stage after AOM/DSS treatment

**Figure 4:**
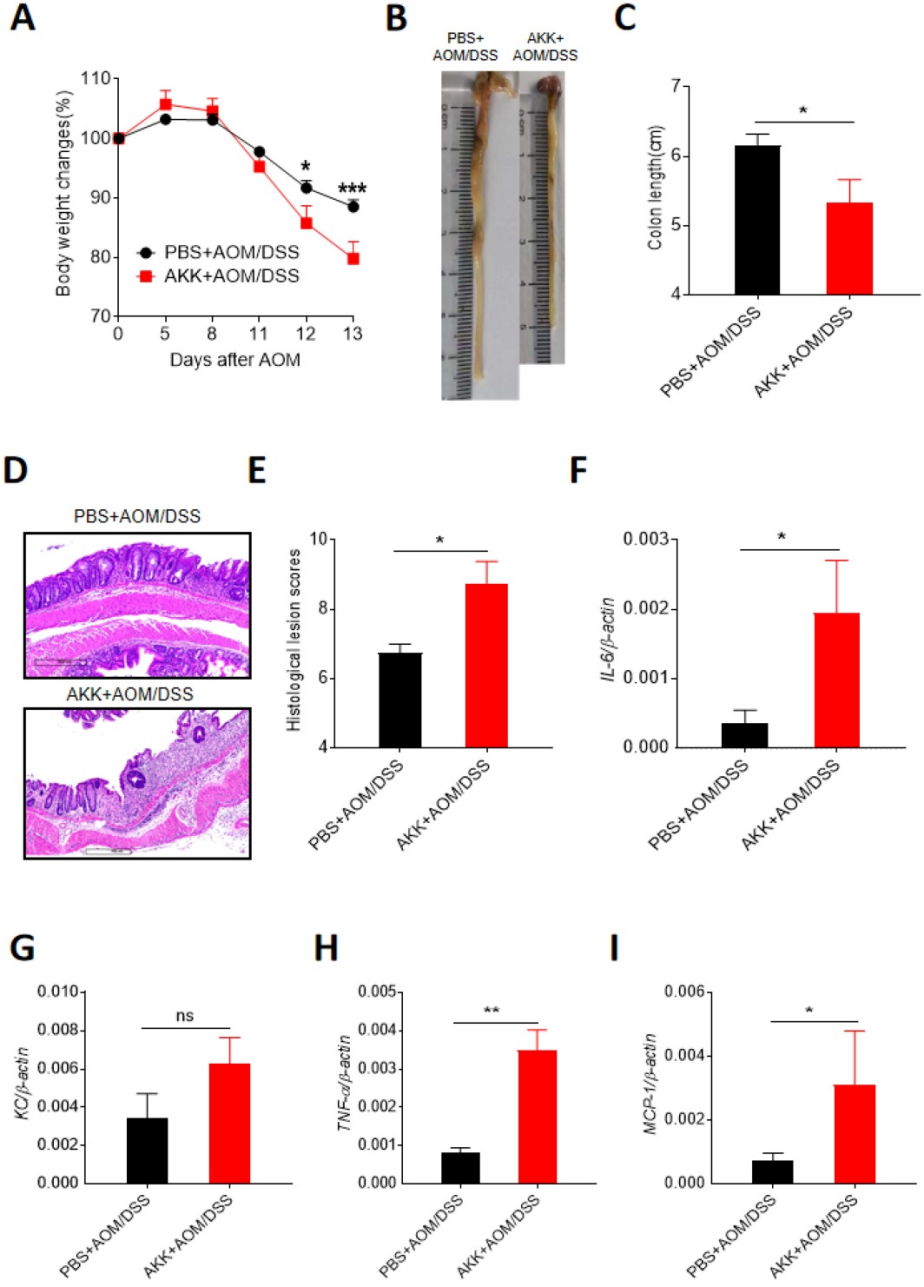
***A. muciniphila* administration exacerbated the inflammation during CRC development.** C57BL/6 mice were orally administrated with *A. muciniphila* or PBS and treated with AOM/DSS. After the first round of DSS treatment (day 14 after AOM injection), the mice were sacrificed to collect the colon tissues. The gene expression of pro-inflammatory cytokines were analyzed by RT-qPCR. **A.** Mouse body weight changes; **B.** Representative colon pictures; **C.** Colon length; **D.** Representative colon H&E staining pictures; **E.** Histological lesion scores; **F.** Colonic *IL-6* gene expression; G. *KC* gene expression; H. *TNF-α* gene expression; I. *MCP-1* gene expression. The data were represented by the Mean ± SEM. PBS+AOM/DSS, n=6; AKK+AOM/DSS, n=4. *P<0.05, **P<0.01, ***P<0.001.ns: no significantly different, A.Two-way ANOVA analysis, C-I. Unpaired student T test. The experiments were repeated twice independently.

To further study the possible role of *A. muciniphila* administration in inducing CRC development. We investigated the colonic changes in 14 days post-AOM injection which was right after AOM treatment and the first round of DSS treatment. Compared to PBS+AOM/DSS group, the mice in AKK+AOM/DSS group lost more body weights (**Figure 4A**), had significantly shorter colon lengths (**Figure 4B-C**), and higher level of histological lesion (**Figure 4D-E**), further suggesting that *A. muciniphila* could increase the host susceptibility to CRC development. Inflammation is an important risk factor in the formation of CRC. We measured the expression levels of multiple classical inflammatory cytokines by RT-qPCR, including Interleukin-6 (IL-6), Tumor necrosis factor alpha (TNF-α), Monocyte chemotactic protein 1 (**MCP-1**), and Keratinocyte chemoattractant (KC). The results showed that the expression levels of all the inflammatory cytokines except KC were elevated in AKK+AOM/DSS group relative to controls (**Figure 4F-I**). These results indicated that *A. muciniphila* could promote the proliferation of intestinal epithelial cells, possibly by aggravating colonic inflammation.

### *A. muciniphila* administration promoted the proliferation of intestinal epithelial cell *in vitro*

**Figure 5:**
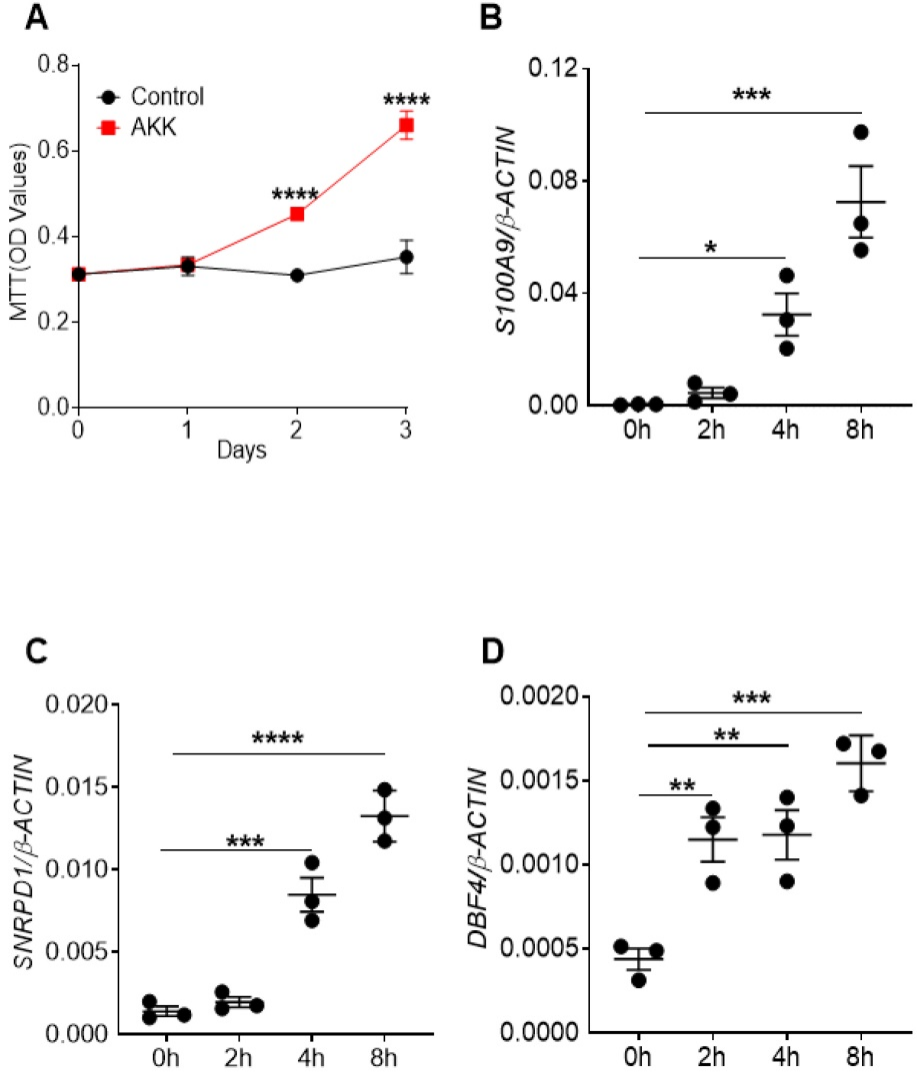
** Cell proliferation rates and gene expression levels of proliferation-related factors after co-culture of *A. muciniphila* and colon epithelial cells.** The colon epithelial cell line SW620 were co-cultured with *A. muciniphila* or PBS for 4 hours and later gentamycine was used to kill extracellular bacteria. The cell proliferation was measured by MTT assay at 0, 1, 2, and 3 days after co-culture. **A.** OD value after co-culture of *A. muciniphila* and colon epithelial cellsThe colon epithelial cell line SW620 was incubated with *A. muciniphila* or PBS. At 0, 2, 4, 6, and 8 hours after the co-culture of colon epithelial cell line SW620 and *A. muciniphila*, cells were washed and and the gene expression of proliferation-related molecules were quantitated by RT-qPCR analysis. **B.** *S100A9* gene expression. **C.** *SNRPD1* gene expression. **D.** *DBF4* gene expression. Data were represented by Mean ± SEM. *P<0.05, **P<0.01, ***P<0.001, ****P<0.0001, A. Two-way ANOVA analysis, B-D. One-way ANOVA analysis followed by Dunnett's *post hoc* test. The experiments were repeated twice independently.

To directly study the possible mechanism of *A. muciniphila* in promoting the proliferation of intestinal epithelial cells, human colon epithelial cell line SW620 cells were co-cultured with or without *A. muciniphila* for 4 hours following gentamycin treatment to kill extracellular bacteria for 3 days. Cell proliferation levels were measured by MTT assay on the 0, 1, 2, and 3 days after co-culture. The results showed the cell proliferation rates of the co-culture group was higher than that of the control group at 2 and 3 days after co-culture (**Figure 5A**). In addition, in this co-culture experiment, cells were collected after gentamicin treatment at 0, 2, 4, 6, and 8 hours respectively. Then RT-qPCR was used to analyze the expression of genes that were associated with cell proliferation. Consistent with the results of MTT assay, the gene expression levels of *S100A9*, *SNRPD1* and *DBF4* were elevated in co-culture group than controls (**Figure 5B-D**). These results suggest that *A. muciniphila* could promote the proliferation of intestinal epithelial cells *in vitro*.

## Discussion

The dysregulation of gut microbiota is considered as a risk factor for CRC. The substantial evidences have proved that alteration of gut microbiota is related to the imbalance in immune response, which is essential for tumor development and progression 20-24. Gut microbiota such as *Escherichia coli*, *Fusobacterium* and *A. muciniphila* play critical roles in the development of CRC through regulating host immune response 25, 26.

*A. muciniphila* is a commensal bacterium mainly colonized in the mucus layer of human intestine, which is associated with intestinal barrier function due to its ability to degrade mucin. Previous studies have shown that the number of *A. muciniphila* in the gut was inversely correlated with diabetes, obesity, and other diseases 27-29. Yet other studies suggest that *A. muciniphila* might promote the development of CRC. However, the live *A. muciniphila*'s effect on CRC development has not been directly investigated.

In our study, mice were orally administered with live *A. muciniphila* or PBS and treated with AOM/DSS to induce CRC. The role of *A. muciniphila* in CRC development was determined by monitoring tumor burden, inflammation status, and proliferation of intestinal epithelial cells. More body weight loss, shorter colon length and greater tumor burden in mice administrated with *A. muciniphila* than controls were observed, indicating their hyper-susceptibility to CRC. Furthermore, more severe tissue damage and higher gene expression of inflammatory cytokines in mice receiving *A. muciniphila* administration were found.

Since *A. muciniphila* can degrade mucins that play a critical role in maintaining mucosal integrity, we speculate that *A. muciniphila* may damage the mucosal barrier, thus leading to invasion of gut microbes and secretion of pro-inflammatory cytokines, ultimately overactive immune response. Excessive pro-inflammatory cytokines combined with the carcinogen AOM accelerated the transformation of normal tissues to dysplastic tissue, inducing more tumor burden in mice administrated with *A. muciniphila*. In addition, *A. muciniphila* promoted the gene expression levels of molecules that modulate proliferation in intestinal epithelial cells. Furthermore, the *in vitro* experiments also showed that the expression of proliferation-related genes in intestinal epithelial cells were up-regulated after cells were co-cultured with *A. muciniphila*. In conclusion, our *in vivo* and *in vitro* results suggest that *A. muciniphila* may promote the CRC development by exacerbating the inflammation and promoting the proliferation of intestinal epithelial cells at the early stage of CRC.

The roles of A. muciniphila in CRC were controversial. There were multiple possible reasons under this discrepancy. First, different bacterial administration procedure such as viability, bacterial numbers, frequency, could all affect the colonization effect of *A. muciniphila* administration and the development of CRC. Unlike a recent study using dead *A. muciniphila* 30, we used live *A. muciniphila* for our administration. Live bacteria could produce various virulence factors, possiblely leading to over-activation of the immune response. Secondly, the gut microbiota, a diverse and dynamic ecological community, can be affected by many factors, such as mouse facilities. The composition of mouse gut microbiota before *A. muciniphila* gavage could significantly affect CRC development. Finally, the role of *A. muciniphila* in different CRC models could be different. In our study, we chose the classical AOM/DSS-induced CRC model, which could be different from spontaneous mouse CRC models such as APC^min/+^ mice. In summary, the influence of *A. muciniphila*'s on CRC development could be different due to different bacterial administration procedure, gut-microbiota composition, and different CRC models.

In conclusion, our study suggests that *A. muciniphila* may promote the development of colitis-associated CRC by aggravating inflammation at the early stage of CRC and enhancing intestinal epithelial cell proliferation. However, further studies were required to dissect the specific mechanism of *A. muciniphila* affecting CRC progression.

## Supplementary Material

Supplementary table.
