# Supplementary material for: Akkermansia muciniphila administration exacerbated the development of colitis-associated colorectal cancer in mice
Source: J Cancer. 2022 Jan 1;13(1):124–33. doi: 10.7150/jca.63578 (PMC8692691; doi:10.7150/jca.63578)
Supplement: Supplementary file 1 — Supplementary table. [file jcav13p0124s1.pdf]

Table S1 List of primers for each gene used in the experiment

Primer names	Primer sequence (5’-3’)
	**Mouse**
**mβ-actin**	(F): CAG CTT CTT TGC AGC TCC TT
	(R): CAC GAT GGA GGG GAA TAC AG
**mIL-6**	(F): TAG TCC TTC CTA CCC CAA TTT CC
	(R): TTG GTC CTT AGC CAC TCC TTC
**mKC**	(F): CTGGGATTCACCTCAAGAACATC
	(R): CAGGGTCAAGGCAAGCCTC
**mMCP-1**	(F): CTT CTG GGC CTG CTG TTC A
	(R): CCA GCC TAC TCA TTG GGA TCA
**mTNF-α**	(F): CCCTCACACTCAGATCATCTTCT
	(R): GCTACGACGTGGGCTACAG
	**Human:**
**hβ-actin**	(F): CAT GTA CGT TGC TAT CCA GGC
	(R): CTC CTT AAT GTC ACG CAC GAT
**S100A9**	(F): GGT GGA AGC ACA GTT GGC A
	(R): GTG TCC AGG TCC TCC ATG ATG
**SNRPD1**	(F): CAG CAT GAA CAC ACA CCT TAA AG
	(R): GCC TCG AAT ACT CAA TGT TTC CA
**DBF4**	(F): AAT AAG ATA CAG TGT CGG GTC CC
	(R): GTC CTT CTG GAA ATT GGG CTC
	**Bacteria:**
Akkermansia	(F): CAGCACGTGAAGGTGGGGAC
muciniphila	(R): CCTTGCGGTTGGCTTCAGAT
**Eubacteria**	(F): ACTCCTACG GGA GGC AGC AGT
**Universal**	(R): ATTACCGCGGCTGCTGGC
